# Spatial computation with gamma oscillations

**DOI:** 10.3389/fnsys.2014.00165

**Published:** 2014-09-09

**Authors:** Ben Engelhard, Eilon Vaadia

**Affiliations:** ^1^Department of Medical Neurobiology, Institute of Medical Research Israel-Canada, The Hebrew University Hadassah Medical SchoolJerusalem, Israel; ^2^Edmond and Lily Safra Center for Brain Sciences, The Interdisciplinary Center for Neural Computation, The Hebrew University of JerusalemJerusalem, Israel

**Keywords:** gamma oscillations, functional topography, temporal synchrony, phase coding, cortical computation

## Abstract

Gamma oscillations in cortex have been extensively studied with relation to behavior in both humans and animal models; however, their computational role in the processing of behaviorally relevant signals is still not clear. One oft-overlooked characteristic of gamma oscillations is their spatial distribution over the cortical space and the computational consequences of such an organization. Here, we advance the proposal that the spatial organization of gamma oscillations is of major importance for their function. The interaction of specific spatial distributions of oscillations with the functional topography of cortex enables select amplification of neuronal signals, which supports perceptual and cognitive processing.

## Introduction

Neuronal oscillations reflect synchronized network activity important for brain computation (Buzsáki, 2006). Such oscillations have been recorded using electrodes placed on the scalp (Berger, 1929), intracranially on the brain surface (Jacobs and Kahana, 2010) and implanted in the brain itself, allowing for recording in both cortical and subcortical structures (Buzsáki and Draguhn, 2004; Schnitzler and Gross, 2005).

In this perspective we focus on oscillations in the gamma band, typically described in the range of 30–70 Hz. Gamma activity has been related to many behavioral conditions including cognitive functions (Herrmann et al., 2010; Siegel et al., 2012). Additionally, clinical interest in this frequency band has increased following studies which have linked abnormal gamma activity with various brain disorders, including Schizophrenia, Autism, ADHD, Alzheimer’s Disease and others (Uhlhaas and Singer, 2006, 2012; Bašar and Güntekin, 2008). Thus, the study of the function and mechanisms of gamma oscillations is significant for basic science as well as for clinical applications. Computationally, gamma-band activity has been hypothesized to reflect the temporally synchronized firing of single units, which could serve as a basis for associations of neuronal ensembles (Gray and Singer, 1989; Singer and Gray, 1995). However, the functional role of gamma activity and the computational means by which it is achieved is still unclear (Palanca and DeAngelis, 2005; Fries, 2009; Wang, 2010; Burns et al., 2011). Here, we propose that gamma activity reflects an oscillatory synchronization process distributed in a spatially-specific manner, which is characterized by precise-time correlation of single neurons that enables coincident firing onto target populations. The flexibility of the proposed computation lies in the interplay between the spatial distribution of the oscillatory process, the amplitude of oscillations, and the modulations of firing rates of tuned neuronal populations.

## Phase-locked firing amplifies efficacy of convergent activity

**Figure 1:**
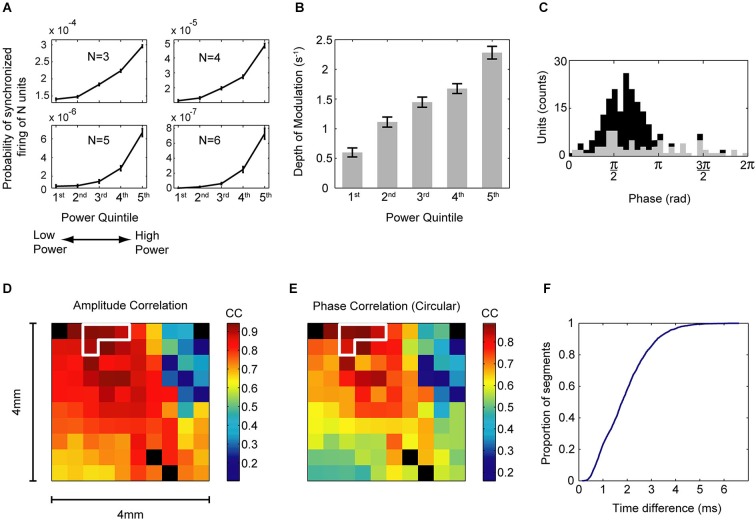
**Relationship between oscillation strength, single neuron synchrony, and oscillatory properties across the cortical space**. **(A)** Probability of N recorded units to fire together in a 5 ms bin with N shown in each frame, for *N* = 3 to *N* = 6. This probability was calculated separately for five periods of increasing power of low-gamma oscillations (30–43 Hz), shown on the *x* axis. Error bars are SEM. The size of synchronized ensembles increased with the amplitude of oscillations. **(B)** Mean ± SEM of the depth of modulation of the cross-correlation histograms for five periods of increasing power of oscillations (30–43 Hz), shown on the *x* axis. Data are from pairs that exhibited significantly increased depth of modulation in segments of high oscillation power. The depth of modulation is a measure of the strength of pairwise neuronal interactions. **(C)** Histogram of the preferred phases of firing for all single units in segments of high oscillation power. Counts of significant preferred phases are marked in black (Raleigh test, *p* < 0.0001 with Bonferroni correction). 63.6% of the 218 single units had a significant preferred firing phase. Note the tight temporal clustering of the preferred phases of firing. **(D)** Mean correlations of the instantaneous amplitude of 30–43 Hz oscillations of LFP between all sites and sites conditioned for an increase in power (circled in white). Data are from one recording day and for segments of high oscillation power. Note the spatial distribution of oscillations (which is not circular) and high correlations near the conditioned sites. The recording array was 4 × 4 mm and had 400 μm interelectrode spacing. Black squares are non-recording electrodes. **(E)** Mean circular correlations between the instantaneous 30–43 Hz phase in all sites and sites conditioned for an increase in power (circled in white). Data are the same as in **(D)**. **(F)** Cumulative distribution of mean absolute time differences between phases of the oscillations in the conditioned sites for segments of high oscillation power. Data are the same as in **(D)**. Note the small time differences for the majority of the segments. Panels **(A–C)** adapted from Engelhard et al. (2013).

Temporal synchrony in spike firing results in an increase in coincident events (Abeles, 1991; König et al., 1996). What would the effect of such synchrony be on target neuronal populations? Evidence suggests that cortical neurons have enhanced sensitivity to synchronous inputs (Abeles, 1982; Azouz and Gray, 2000, 2003; London and Häusser, 2005). Accordingly, by coincidence detection, synchronous signals would be amplified (König et al., 1996). In fact, this phenomenon has been reported in thalamocortical circuits, where weak thalamic projections to cortex induce strong feed-forward excitation by way of synchronous activity of convergent inputs (Bruno and Sakmann, 2006). Thus, temporal synchrony can amplify the effect of neuronal activity without requiring an increase in the firing rate of the inputs. How is this synchrony achieved in the presence of oscillations? There is evidence that when cortical networks enter a state characterized by gamma oscillations, neurons fire in a phase-locked manner, hence increasing the temporal synchrony between them (Singer and Gray, 1995). Further, we have recently shown that the relationship between the strength of gamma oscillations in the local field potentials (LFP) and the strength of precise-time spike synchrony is quantitative (Engelhard et al., 2013; see Figure 1); namely, periods with increased amplitude of gamma oscillations are characterized by stronger pairwise interactions as well as by an increase in the size of precisely synchronized assemblies. Thus, during a strong oscillatory state of the network (in the gamma band) the increase in temporal synchrony would amplify the signals produced by neurons in the oscillating region as long as their output converges onto target populations.

## Oscillation strength and precise-time synchrony

Consider two cortical sites where there is need for temporal synchronization of spike-firing in both sites. What conditions are required for this synchronization by an oscillatory mechanism to take place? First, since the level of temporal synchronization depends on the strength of the oscillations, oscillations in both sites should increase simultaneously. Thus we would expect a correlation between the amplitude of field oscillations in both sites. Second, since the spikes are phase-locked to the oscillations, there needs to be phase coherence (or correlation) between the oscillations in both sites. We also expect the phase lag between oscillations to be small, (for nearby sites) or at least coordinated to optimize the required effects of the sites on the target neuronal population. Third, there should be a tight clustering of the preferred firing phases of neurons in both sites, so that the temporal coincidence of firing is maximal. The second and third conditions constrain the preferred firing time of neurons to be in tight coordination, which is important to reduce the detrimental effect of the temporal jittering noise of spiking around the preferred firing phase.

There is evidence for these three conditions in cortex. Amplitude correlations of gamma activity have been found in distributed sites during a visual comparison task (Bruns et al., 2000). Zero-lag coherent oscillations have been found in relatively nearby as well as distant sites (König et al., 1995). In our study, where monkeys were operantly trained to increase the power of gamma oscillations of the LFP in a subset of four electrodes out of a 96 electrode-array, we found both a strong amplitude and phase correlation between oscillations in the conditioned sites, and small phase differences between them (Engelhard et al., 2013; see Figure 1). The small scale of our array (400 μm interelectrode distance) complements the larger scale findings and alludes to the pervasiveness of this phenomenon. Additionally, we found tight clustering of the preferred phases of firing, so that most neurons preferentially fired in the falling flank of LFP, as has been shown elsewhere (Murthy and Fetz, 1996; Denker et al., 2011).

The data reported in Hasenstaub et al. (2005) further supports this view. They showed that although fast spiking interneurons and regular spiking cells have a different mean overall preferred firing phase, both these populations have a relatively narrow range of preferred phases of firing in the gamma cycle. The standard deviation of the preferred firing phases for regular spiking cells was 2.4 ms; such temporal clustering in the preferred firing phase across the population would enable coincident firing for neurons in sites characterized by high oscillation amplitude, as proposed here. Another study (Womelsdorf et al., 2012) showed similarly tight clustering in the primary visual cortex of awake monkeys.

It is important to note that the type of computation-by-coincidence described here does not require a perfectly regular unfolding gamma phase, as the amplification effect on target populations can be achieved whenever coincident firing occurs, regardless of the regularity between coincident episodes. This is significant because cortical gamma oscillations may exhibit variability in their phase distribution across cycles (Burns et al., 2011).

Finally, if the level of oscillations is involved in computations related to cognitive tasks, we would expect to observe a relationship between oscillation strength and behaviorally relevant parameters. Such relationships have been widely observed in different tasks and brain areas. For example, in the primary visual cortex, gamma power was shown to depend on the level of stimulus contrast (Logothetis et al., 2001; Henrie and Shapley, 2005). In the primary somatosensory cortex, gamma power changed in relation to different levels of subjective pain intensity (Zhang et al., 2012). In the posterior parietal cortex, there were changes in gamma power corresponding to the planning of different movement behaviors (Scherberger et al., 2005).

## Spatial distribution of oscillations

In this section we first address the benefits of spatially-based computations in cortex; we then ask which experimental findings would be suggestive that gamma oscillations are involved in such computations, and proceed to review the presence of these findings in the literature.

Above, we discussed how precise-time synchrony can amplify the signal of the synchronized neurons, and showed that when the network enters an oscillatory state such synchrony can be achieved. However, in order to provide a substrate for effective computations this amplification should be specific to task-related neuronal assemblies. The spatial distribution of oscillations could be a mechanism to enable specific amplification output signals in particular cortical areas. The plausibility of this hypothesis stems from the finding that the spatial pattern of gamma oscillations is tightly linked to the spatial pattern of precise-time synchrony (Engelhard et al., 2013).

Functional topographic organization is a prominent feature in sensory cortex, and reflects the orderly convergence of afferents to adjacent sites (Mesulam, 1998). In the motor cortex, apart from the gross homunculus (Penfield and Boldrey, 1937), functional mapping is much less clear (Schieber and Hibbard, 1993; Ben-Shaul et al., 2003; Rathelot and Strick, 2006; but see Georgopoulos et al., 2007). In fact, the wide intermingling of cortico-muscular neurons in different muscle-related areas has led to the view that functional diversity across the cortical space is conducive to the planning and production of movements involving different muscle synergies (Rathelot and Strick, 2006). This view has also garnered support from electrical micro-stimulation studies showing that natural movements can be evoked by site-specific stimulation (Graziano et al., 2002). Thus, in many cortical areas, focal activity of nearby neurons seems to be functionally relevant. A mechanism that exploits these relations by activating spatially-defined populations that are behaviorally congruent and with sufficiently rapid activation timescales would therefore be practicable for perception, motor functions and cognitive processing.

**Figure 2:**
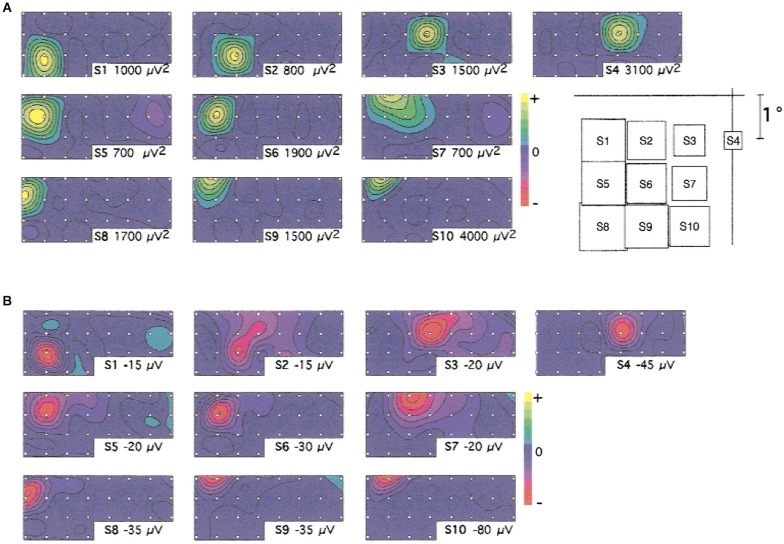
**Spatial distribution of gamma activity across the cortical space in V1. (A)** Topographical distribution of the sustained gamma oscillations (30–70 Hz) at the different stimulus locations tested (indicated on the right). **(B)** Topographical distribution of the first negative component of the evoked potential, for the same stimuli, at 64 ms. Note the stimulus-dependent spatial distribution of the gamma oscillations and evoked component. ECoG was recorded using an implanted array with 3 mm interelectrode spacing. Figure reproduced with permission from Rols et al. (2001).

If gamma oscillations are related to spatial computations in cortex, we would expect experimental evidence for stimulus (or stimulus (or task) dependence of the spatial distribution of oscillations across the cortical space with oscillations that are functionally relevant to the task or stimulus. These phenomena have been reported in cortex. The stimulus dependence of the spatial distribution of oscillations was clearly evidenced in both V1 and V4 for visual stimuli (Rols et al., 2001; see Figure 2). Further, the topography of oscillations followed the retinotopic cortical map. Similarly, gamma activity related to attention was found to be localized in regions responsive to the particular stimulus in V4 (Taylor et al., 2005). Such stimulus- or task-specific spatial distributions have also been found in a short-term memory task (Tallon-Baudry et al., 1998) and during presentations of coherent visual patterns (Lutzenberger et al., 1995). An electrocorticography (ECoG) study found stimulus-specific spatial distributions of gamma activity in response to visually presented letters (Jacobs and Kahana, 2009). In the auditory cortex of monkeys, stimulus-dependent gamma oscillations have been described with spatial configurations that depend on the specific frequency response properties of the recorded sites (Brosch et al., 2002).

Further support for this idea comes from a study which showed that specific thalamic stimulation evokes gamma oscillations in modality-specific cortical areas (Macdonald et al., 1998). This demonstrates the presence of thalamocortical circuits capable of inducing specific spatial distributions of gamma oscillations.

## Neuronal diversity and tuned populations

While in theory the described spatial computation would work well in a cartoon cortex with perfect topography, in actuality there is a wide diversity of function in cortex, even in areas that exhibit a clear functional topography (Ringach et al., 2002; Rothschild et al., 2010). This diversity is compounded in areas where such topography is much less clear, such as the primary motor cortex (Rathelot and Strick, 2006). Thus, there needs to be a mechanism to enable differentiation between nearby neurons with widely differing functions. We propose that neuronal tuning via modulation of their firing rate, which is perhaps the most widely accepted mechanism for neural coding (Adrian and Zotterman, 1926; Shadlen and Newsome, 1994), could be such a mechanism. The presence of oscillations does not abolish the tuning of firing rates (Friedman-Hill et al., 2000; Liu and Newsome, 2006; Ardid et al., 2010). In fact, there is evidence that such tuning might even be enhanced in the presence of oscillations (Paik et al., 2009; Womelsdorf et al., 2012). Therefore, in the same volume of space, neurons coding for the currently relevant function would increase their firing rate, and because of phase-locking the coincidence of their spikes would amplify the signal they emit, while neurons coding for other functions would decrease their firing and would not avail themselves of this effect. Such a scheme makes further sense considering that most cortical neurons fire at low rates (Abeles et al., 1990; Shafi et al., 2007; Hromádka et al., 2008) and thus spurious coincidence firing will hence be small. Viewed in this way, oscillatory spatial computation is not an independent computational process but rather works in conjunction with previously found coding schemes such as rate coding, in order to enhance the temporal precision of functionally congruent populations.

## Relationship with phase-of-firing coding

Here we propose a model that does not require individual coordination of the timing of each neuron according to task demands. An earlier, more complex model, termed phase-of-firing (PoF) coding assumes that information is present in the phase at spike firing of each neuron (Perkel and Bullock, 1968; Hopfield, 1995; Wang, 2010). Such coding has been found mostly at lower frequencies than gamma, for example, theta oscillations in the hippocampus (Huxter et al., 2008) or alpha oscillations in the auditory cortex (Kayser et al., 2009). One study described PoF coding in the primary visual cortex (Montemurro et al., 2008). They reported that such coding, at low frequencies of oscillations, carried up to 54% more information about natural stimuli than spike counts alone. The information about the stimuli decreased for higher frequencies and was negligible for frequencies higher than 24 Hz. However, there have also been descriptions of PoF coding for gamma-band frequencies in cortex. For example, Havenith et al. (2011) reported that different sequences of neurons fired in response to different stimuli, and that these sequences were related to 20–60 Hz oscillations. Other descriptions of gamma-band PoF coding have been reported as well (Masquelier et al., 2009). We proceed to examine the relationship between PoF coding and the proposal outlined here.

Clearly, PoF coding enables a higher information rate, as the information transmitted by the phase of each spike allows for a much richer representation with the same number of spikes. On the other hand, the single-phase spatial computation described here is more robust to noise since temporal jittering of spike timing would not lead to erroneous encoding. Rather, it implies less efficacious coincidences, which can be easily remedied by increasing the number of neurons involved, or the amplitude of oscillations (which is tightly related to the phase-locking and hence the temporal specificity of firing). For PoF coding, we expect that as the oscillation frequency increases, the detrimental effect of noise will increase as well, because as the oscillation cycle shortens each temporal jittering error reflects a larger phase error. Additional difficulties related to PoF coding at high frequencies have been considered as well (Montemurro et al., 2008; Wang, 2010). Thus, the two coding schemes can be seen as complementary: in low noise or low frequency regimes, PoF coding may provide powerful, high-throughput coding, while in the presence of increased noise or higher frequencies, single-phase spatial computation can provide a robust alternative. In this context is worth noting that it might be hard to disassociate between PoF and single-phase spatial-computation by standard experimental measures. For instance, a realistic example of spatial computation might not present exactly a zero-phase difference across all sites; the generation of the spatial distribution of oscillations may involve that oscillations in some sites start earlier than in others, resulting in small phase differences across sites. Since these differences depend on the spatial distribution of oscillations, they are stimulus dependent. Thus, repeatable, stimulus-dependent differences in phase will result in stimulus-dependent differences in the timing of neuronal firing for neurons in different sites, which is commonly considered a characteristic of a PoF code. To differentiate between the two coding schemes we propose the following experiments and data analysis procedures: (1) recordings of spiking activity with high spatial resolution should be performed, after which a spatial analysis can be effectuated to determine whether the differences in timing of neuronal firing can be accounted for by the spatial location of the neurons. A strong relationship between spatial location and phase of firing would support the spatial computation hypothesis; (2) simultaneous recordings from multiple connected sites can be used to assess whether the differences in timing of firing (in the upstream neurons) have an effect on the firing patterns and coding capabilities of downstream neurons. If upstream neurons evidence multiple preferred firing phases which have a distinct effect on the downstream population, this can be construed as support for PoF coding.

## Mechanism

Although the mechanism of generation and maintenance of cortical gamma oscillations is still subject to intensive research, there is converging evidence that inhibitory subpopulations are critically involved in the formation of this oscillatory process (Buzsáki and Wang, 2012). Networks of inhibitory interneurons have been shown to mediate ~40 Hz oscillations *in vitro* (Whittington et al., 1995); rhythmic activation of fast-spiking inhibitory neurons (but not regular-spiking excitatory neurons) is necessary for the generation of these oscillations in cortex *in vivo* (Cardin et al., 2009). One study reported a relationship between the ability of humans to discriminate between different orientations and the concentration of GABA, as well as the frequency of stimulus induced gamma oscillations in V1 (Edden et al., 2009). These results imply that studying the spatial connectivity patterns and spatial network properties of inhibitory subpopulations is an essential step in understanding the mechanism for generation and maintenance of specific spatial distributions of oscillations. The inhibitory network may play a key role in selecting between the different oscillatory spatial configurations according to task demands.

## Local vs. distant remote spatial computation

Gamma oscillations have frequently been described with regard to local processing (reviewed in Donner and Siegel, 2011). Spatial modulation of coincident firing is a plausible mechanism to support such processing. However, coherent gamma oscillations for spatially-remote regions have been described as well (Engel et al., 1991; Fries et al., 2001; Doesburg et al., 2008). Thus, computationally relevant spatial distribution patterns of oscillation could in principle involve spatially discontinuous topologies, even between hemispheres (Engel et al., 1991; König et al., 1995; Gregoriou et al., 2009).

### Limitations and future prospects

Most of the evidence we have described is the outcome of recordings from the neocortex. There may be characteristic differences in neuronal firing patterns in other brain areas where the oscillatory spatial computation described here is less likely. For example, in the hippocampus, pyramidal neurons typically have much weaker phase-locking to gamma oscillations, and population histograms of preferred phases are much broader, allowing for the separation of the pyramidal population into clusters based on their preferred firing phase (Senior et al., 2008). Thus, we expect a decrease in the precision of coincident firing under these circumstances, where the more prominent feature appears to be phase-coding with respect to theta oscillations (Buzsáki, 2002).

We have exclusively analyzed correlations arising from an oscillatory process in the gamma range, but in cortex there is evidence for both oscillatory and non-oscillatory modes of correlated activity (Eckhorn, 1994; Baker et al., 2001). The current framework does not account for non-oscillatory synchrony and accordingly does not aim to encompass the full gamut of possible modes of neural coordination but rather provide a simple explanation as to the role of oscillatory processes in the gamma range with regard to functional neuronal patterns.

Finally, it is clear that compared to the vast array of studies on gamma oscillations, there are few systematic spatial analyses of oscillatory properties. This state of affairs is likely to change as result of technological advances such as dense microelectrode arrays, high-resolution imaging and spatially-precise optogenetic manipulations. Future experiments using these methodologies for simultaneous recordings from identified neuronal populations will be better equipped to characterize the functional importance of the spatial properties of oscillations and single unit synchrony patterns.

## Conflict of interest statement

The authors declare that the research was conducted in the absence of any commercial or financial relationships that could be construed as a potential conflict of interest.
